# COVID-19: Detecting Government Pandemic Measures and Public Concerns from Twitter Arabic Data Using Distributed Machine Learning

**DOI:** 10.3390/ijerph18010282

**Published:** 2021-01-01

**Authors:** Ebtesam Alomari, Iyad Katib, Aiiad Albeshri, Rashid Mehmood

**Affiliations:** 1 Department of Computer Science, Faculty of Computing and Information Technology, King Abdulaziz University, Jeddah 21589, Saudi Arabia; EAlomari0011@stu.kau.edu.sa (E.A.); IAKatib@kau.edu.sa (I.K.); AAAlbeshri@kau.edu.sa (A.A.); 2 High Performance Computing Center, King Abdulaziz University, Jeddah 21589, Saudi Arabia

**Keywords:** COVID-19, coronavirus, machine learning, big data, social media, apache spark, Twitter, Arabic language, distributed computing, smart cities, smart healthcare, smart governance, Triple Bottom Line (TBL)

## Abstract

Today’s societies are connected to a level that has never been seen before. The COVID-19 pandemic has exposed the vulnerabilities of such an unprecedently connected world. As of 19 November 2020, over 56 million people have been infected with nearly 1.35 million deaths, and the numbers are growing. The state-of-the-art social media analytics for COVID-19-related studies to understand the various phenomena happening in our environment are limited and require many more studies. This paper proposes a software tool comprising a collection of unsupervised Latent Dirichlet Allocation (LDA) machine learning and other methods for the analysis of Twitter data in Arabic with the aim to detect government pandemic measures and public concerns during the COVID-19 pandemic. The tool is described in detail, including its architecture, five software components, and algorithms. Using the tool, we collect a dataset comprising 14 million tweets from the Kingdom of Saudi Arabia (KSA) for the period 1 February 2020 to 1 June 2020. We detect 15 government pandemic measures and public concerns and six macro-concerns (economic sustainability, social sustainability, etc.), and formulate their information-structural, temporal, and spatio-temporal relationships. For example, we are able to detect the timewise progression of events from the public discussions on COVID-19 cases in mid-March to the first curfew on 22 March, financial loan incentives on 22 March, the increased quarantine discussions during March–April, the discussions on the reduced mobility levels from 24 March onwards, the blood donation shortfall late March onwards, the government’s 9 billion SAR (Saudi Riyal) salary incentives on 3 April, lifting the ban on five daily prayers in mosques on 26 May, and finally the return to normal government measures on 29 May 2020. These findings show the effectiveness of the Twitter media in detecting important events, government measures, public concerns, and other information in both time and space with no earlier knowledge about them.

## 1. Introduction

The level of digital and physical connectedness of today’s societies has never been seen before. We are able to see and talk to people on the other side of the planet as if they are with us. We are able to control machines in the farthest continents using our smartphones. We are able to physically travel across the world in a day. We travel a lot to distant lands and frequently share gifts and viruses with each other. 

Unfortunately, the COVID-19 pandemic has exposed the vulnerabilities of this unprecedentedly connected world. The COVID-19 pandemic, or coronavirus pandemic (COVID-19 is the name of the disease), is caused by the virus SARS-CoV-2 (Severe Acute Respiratory Syndrome CoronaVirus 2) [1]. As of 19 November 2020, over 56 million people have been infected with nearly 1.35 million deaths, and the numbers are growing [1]. Social, economic, and environmental sustainability has been severely affected throughout the world. There is a growing consensus that the post-pandemic societies and world may take a different course for living, work, education, and other spheres of life. If (partial or full) remote working, businesses, and education are to become the norm, many people may choose to move and live in suburban or rural areas, and this will shift the course of urbanization.

There is a need to understand what is happening around the world during and after the pandemic, what measures are being taken to fight the pandemic by the government and authorities, what the needs of the people are, and what people’s concerns and priorities are, etc. This information can help us to understand the implications of various pandemic measures (e.g., social isolation), manage the pandemic, address people’s concerns, understand the impact of various policies for the post-pandemic future, and more. 

Traditional methods of data collection and analysis using surveys and other means cannot capture such timely and large-scale data, alongside them having other disadvantages. Researchers in recent years have increasingly used social media including Twitter to study different issues in many application domains and sectors [2,3,4,5]. Naturally, social media has also been used during the COVID-19 pandemic times even more so, because the use of social media and virtual platforms by the public has increased due to social isolation and reduced mobility. A detailed literature review has revealed that the state-of-the-art research on social media analytics for COVID-19-related studies is limited. Many more studies are needed to improve the breadth and depth of the research on the subject in several aspects (Section 2 elaborates the research gap, novelty, and contributions of our work).

### 1.1. Description of the Proposed Work

In this paper, we propose a software tool comprising a collection of machine learning and other methods for the analysis of Twitter data in Arabic with the aim to detect government pandemic measures and public concerns during the COVID-19 pandemic. The methods used in the tool include an unsupervised Latent Dirichlet Allocation (LDA) topic modeling algorithm, natural language processing (NLP), correlation analysis, and other spatio-temporal information extraction and visualization methods. The tool was built using a range of technologies including MongoDB, Parquet, Apache Spark, Spark SQL, and Spark ML. The tool comprises five software components (see Section 3). The Data Collection and Storage Component (DCSC) uses various search queries and geo-coordinates to collect data using Twitter REST (Representational State Transfer) API (Application Programming Interface) and stores it using MongoDB and Apache Spark DataFrame (DF), a distributed data collection organized into named columns. The Data Pre-Processing Component (DPC) removes noise from the text and provides cleaned, normalized, and stemmed tokens. The Measures and Concerns Detector Component (MCDC) uses an unsupervised LDA model to cluster the tweets and detect government and public measures and concerns. The correlations in data are also computed here. The Spatio-Temporal Information Component (STIC) performs spatial and temporal analysis by extracting the date, time, location, and other information from the tweets. The Validation and Visualization Component (VVC) visualizes the results spatially and temporally using maps and other tools and validates the detected measures and concerns using internal or external sources such as news media. The Twitter dataset used in this specific study comprises 14 million tweets. It was collected using the Twitter API from 1 February 2020 to 1 June 2020 for the Kingdom of Saudi Arabia.

The software developed for this work is part of the tool Iktishaf [6,7,8,9] that we have been developing for the last few years. Earlier work on this tool has focused mainly on mobility-related event detection using supervised learning. We have also developed other tools for big data social media analytics in healthcare [10], logistics [11,12], and public opinion mining for government services [13]. These works have used Twitter data in Arabic or English.

### 1.2. Findings

We formulate and analyze the findings of this paper from three relationship perspectives: information-structural, temporal, and spatio-temporal. 

**Information-Structural Perspective:** Using the tool, in terms of the information-structural (or subject matters) perspective, we have detected 15 government pandemic measures and public concerns (quarantine, loan, salary, mobility, etc.) and have grouped them into six macro-concerns (economic sustainability, social sustainability, contain the virus, etc.). For the **pandemic measures** implemented by the Saudi government in relation to the COVID-19 pandemic, we detect curfew and restrictions on mobility in the country, quarantine and fines, restrictions on praying in the mosques, campaigns to stay home, COVID-19 prevention, and cleaning services provided to curb the coronavirus spread. For **economic sustainability**, we detected that the government provided financial incentives including loans and private-sector salaries. Businesses increased offers to increase their sales. People moved to or increased their online economic activities, such as the activities related to prize draws for income earnings. For health, well-being, and **social sustainability**, we detected that blood donation and treatment at hospitals have been a major cause of concern. People also actively talked about the new number of cases. The **daily livelihood** issues in Saudi Arabia include five daily congregational prayers at the mosques that were suspended by the government. This was a major concern because praying five daily prayers in congregations is compulsory in Islam (with certain exceptions). People usually pray in mosques in congregations, standing close to each other and aligning shoulders and ankles with the person on the right and left, which is risky in the pandemic situation. People also increased in supplications for the safety of people. Roads were found to be empty or with abnormally low traffic during the corona times, and this was also vigorously discussed. A significant reduction in mobility was noted across the country that was related to **environmental sustainability**, health, and well-being due to the reduction in traffic congestion and air pollution. The detected events in Kingdom of Saudi Arabia (KSA) are also aligned with **international concerns**, such as various lockdown measures [14], reduced mobility [15], reduction in blood donations [16], financial difficulties and related government incentives [17,18], and worries related to returning to normal times [19].

**Figure 1:**
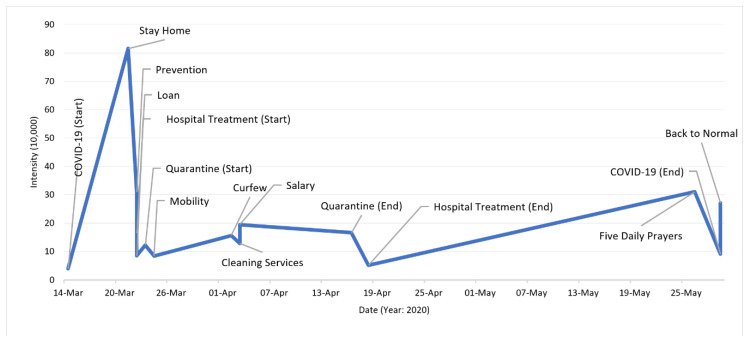
The timeline of some of the detected government pandemic measures and public concerns.

**Temporal Perspective:** Regarding the temporal perspective of the various pandemic-related events within the time period of the dataset (1 February 2020–1 June 2020), we are able to see timely relationships in the progression of various events. Figure 1 shows the timeline of some of the detected government measures and public concerns. Some of these events in the Twitter activity that remained high for a period are shown with their start and end times. The earliest detected events in the data are related to virus infection, prevention, curfew, and stay home. Between **mid-March 2020** and the **end of May** (with some intermittent gaps), people also increased their Twitter activity related to the virus infection concern (spread of coronavirus and the increase in the number of cases). The curfew (7 a.m.–6 p.m.) in Saudi Arabia was ordered firstly on **22 March** and applied from the next day. The events related to loans were detected with the highest peak on **22 March** (Saudi Arabian Monetary Agency (SAMA) announced it on the same day [20]). The activities on Twitter related to the quarantine event (we use “measure”, “concern”, and “event” interchangeably, as appropriate) remained high during **the initial period** of the curfew to around **mid-April**. The “No Mobility” event (empty roads) was vigorously discussed on **24 March**, two days after the curfew was ordered. The curfew situation and the reduction in government services along with people’s fear of getting infected by the virus had caused a reduction in blood donations and blood supplies. The activity on this topic requesting blood donations was seen to be increased from **late March** to **mid-April**. On **2 April**, a 24 h curfew was enforced in Makkah and Medina (the two holiest cities in KSA and the Muslim world), which stirred heavy Twitter activity. The events for the salary events were detected on the **3 April** the day King Salman of Saudi Arabia ordered to contribute towards 60% of the salaries of Saudi private-sector employees with a financial incentive of 9 billion Riyals in total (this was verified through external sources [21]). A peak activity related to the Five Daily Prayers concern was found on **26 May** when the Ministry of Interior announced that they would allow daily prayers to be held in all mosques of the Kingdom (except the mosques in Makkah city). Finally, we detected the “Back to Normal” government measure on **29 May 2020**. 

**Spatio-Temporal Perspective**: We extracted location information using different approaches including tweet text and hashtags, geo-coordinate attributes, and user profiles. We were able to detect important events in over 50 cities around the kingdom with major activities related to COVID-19 cases, curfew, etc., in the Makkah, Riyadh, and Eastern provinces. 

We validated the detected government measures and public concerns and their spatial and temporal nature through external validation by searching online news media or through internal validation by checking tweets. These findings show the effectiveness of the Twitter media in detecting important events, government measures, public concerns, and other information in both time and space with no earlier knowledge about them. 

The organization of the paper is as follows. Section 2 reviews the related works and elaborates on the research gaps. Section 3 explains our methodology and the design of the tool. Section 4 discusses the results and analysis. Section 5 gives the conclusions and directions for future work. 

## 2. Literature Review

Smart cities and societies are driven by the need to provide highly competitive, productive, and smarter environments through the innovation and optimization of urban processes and life [22,23]. Artificial intelligence has taken us by storm [24], and has led to the emergence of concepts such as artificially intelligent cities [25]. A key to providing smartness for emerging urban and rural environments is to continuously sense and analyze these environments and make timely and effective decisions [6,10,24,26]. Social media analysis using machine learning has become a key method to provide the pulse for sensing and engaging with the environments [6] and is expected to provide smarter solutions for our fight against COVID-19 and future pandemics as well as during peace times. 

We review here the literature relevant to the topic of this paper, which is the detection of COVID-19-related public concerns from social media (big) data in the Arabic language using machine learning, specifically the LDA topic modelling method. Firstly, in Section 2.1, we provide a background on the pre-COVID-19 use of social media in various application domains. Subsequently, we review in Section 2.2 the works about COVID-19 analysis that have used social media data without limiting the reviewed works to any analysis method or a language. In Section 2.3, we review the works about COVID-19 analysis and social media that have specifically used topic modelling for analysis purposes; these works are not limited to any language. We focus on the Arabic language in Section 2.4 and review the works related to COVID-19 analysis that use Twitter data. Finally, Section 2.5 discusses the research gap.

### 2.1. Use of Social Media in Research (Pre-COVID-19)

Digital societies could perhaps be characterized by their increasing desire to express themselves and interact with others, and this is done through various digital platforms such as social media. It is reported that roughly 58% of the global “eligible population” (70% of the eligible population in 100 countries around the world) uses social media [10,27]. Social media could provide a two-way communication channel for individuals, governments, businesses, and others to engage with their friends, communities, stakeholders, etc. [10]. The traditional methods of data collection and analysis using surveys and other means cannot capture such timely and large-scale data, alongside them having other disadvantages. Researchers in recent years have increasingly used social media including Twitter to study different issues in many application domains and sectors, and this trend has been ramping up in COVID-19-related research and other studies. Social media and Internet of Things (IoT) provide the pulse for sensing and engaging with the environments [10]. Sentiment analysis, or opinion mining, that utilizes social and other textual media is a vital tool in natural language processing (NLP), defined as “the field of study that analyzes people’s opinions, sentiments, evaluations, appraisals, attitudes, and emotions toward entities such as products, services, organizations, individuals, issues, events, topics, and their attributes” [10,28]. Many of the notable works on sentiment analysis rely on machine learning and social media, with applications in logistics and urban planning [12,25,29,30,31]; categorizing tweets about road conditions into useful, nearly useful, and irrelevant complaint tweets [2]; identifying sources of noise pollution [32]; extracting traffic-related information from tweets [3]; general and traffic-related event detection [6,9,11,33,34,35]; public opinion mining for government services [13]; detecting health-related topics from the stream of tweets (without aiming to detect a particular illness) [36]; tracking the side effects of certain medications [37]; the detection of top symptoms, diseases, and medications and related awareness activities [10]; tracking flu infections on Twitter [38], influenza surveillance from social media data [39,40,41]; and many more.

### 2.2. COVID-19 and Social Media (General)

We review here the works about COVID-19 analysis that have used social media data without regard to any modelling method or a language. Singh et al. [42] analyzed tweets about coronavirus in different languages including English, French, German, Italian, and others. Furthermore, they have performed spatiotemporal analysis of the data. They focused on three countries, which are the United States, Italy, and China, and showed the time series of tweets and the daily confirmed COVID-19 cases. They found that the countries that had a higher number of COVID-19 cases also had a higher number of tweets about COVID-19. Gencoglu [43] applied supervised classification to capture COVID-19-related discourse during the pandemic. They collected around 26 million tweets using Twitter streaming API with keyword filtering. They trained classifiers using k-nearest neighbor, logistic regression, and support vector machine (SVM) to classify the tweets into 11 categories including donate, prevention, reporting, share, speculation, symptoms, and others. For training the machine learning classifiers, they utilized two annotated datasets of questions and comments related to COVID-19. The dataset consisted of several languages, including English, French, and Spanish, and was generated by native-speaker annotators based on an ontology. Then, they employed language-agnostic BERT (Bidirectional Encoder Representations) sentence embeddings to obtain a pre-trained model. To extract embeddings, they used the TensorFlow framework on a 64-bit Linux machine with an NVIDIA Titan Xp GPU. They found that Twitter activity increased due to the increase in the spread of COVID-19 across the world. 

Several other works on the use of social media for COVID-19 analysis have been reported. These include studies on American and Chinese peoples’ views on COVID-19 [44], the mood of Indian people during the pandemic [45], the spread of anti-Asian hate speech [46], the political tension between Brazil and China [47], and the identification of emotional valence and predominant emotions [48]. Moreover, others have looked into modelling social media data for COVID-19-related analysis to study the spread of misinformation about the coronavirus [49,50,51], discovering political conspiracies in the U.S. that were posted by Twitter automated accounts during the COID-19 outbreak [52], identifying the causal relationship of the daily Twitter activity and sentiments during the pandemic [53], and studying the frequency of the phrases “Chinese virus” and “China virus” before and after the outbreak in the United States [54]. 

None of the works reported in this subsection have a focus or methods similar to our research reported in this paper. None of them have used the distributed big data computing framework Apache Spark. The discussed works did not support social media in the Arabic language, which, as mentioned earlier, has its own challenges, particularly since it is not based on the Latin script. Moreover, the size and period of the used data are also different. 

### 2.3. COVID-19 and Topic Modeling

We review here the works about COVID-19 analysis using social media that have specifically used topic modelling as the modelling method. These works are not limited to any language. Liu et al. [55] studied the role of the Chinese mass media during the COVID-19 crisis using news articles from the WiseSearch database. They applied LDA and extracted 20 topics and then classified them into nine themes. The topics include prevention and control policy, prevention and control measures, medical affiliation and staff, epidemiologic study, and others. The themes include confirmed cases, prevention and control procedures, medical treatment and research, detection at public transportation, and others. Kaila and Prasad [56] applied LDA analysis and found the topics related to the coronavirus from 18,000 tweets. Besides, they applied sentiment analysis and found that most of the tweets were negative. Abd-Alrazaq et al. [57] identified twelve topics from 167,073 tweets, collected for the period 2 February 2020 to 15 March 2020, using LDA and grouped them into four themes: the origin of COVID-19, the source of the novel coronavirus, the impact of COVID-19 on people and countries, and the methods for decreasing the spread of COVID-19. Then, they used a simple string-matching technique to find tweets that contain the selected keywords of the topics. Additionally, they calculated the interaction rate for each topic after calculating the sentiment score and the number of retweets, likes, and followers for each topic. None of the works discussed in this paragraph have applied temporal or spatial analysis, supported social media in the Arabic language, or used distributed big data computing platforms such as Apache Spark.

Med [58] collected 94,467 posts from the Reddit website in the period between 3 March and 31 March. Then, they applied LDA and found 50 topics, 10 of them were assigned to one of the following categories: public health measures, daily life impact, and sense of pandemic severity. After that, they measured daily changes in the frequency of topics. Ordun et al. [59] applied keyword analysis to find the most frequent words. They analyzed around 5.5 million tweets in different languages that are based on Latin script. Arabic, Chinese, and other languages that are based on non-Latin scripts were not included. They used term-frequency inverse-document-frequency (TF-IDF) and defined the max_features to 10,000. In addition, they performed topic modeling and identified twenty topics using the default parameters of the Gensim LDA MultiCore model. For each topic, they extracted the top twenty terms and used the first three terms to label the topic. Further, they used Uniform Manifold Approximation and Projection (UMAP) to visualize how the 20 topics grouped together. Additionally, they performed a temporal analysis to examine the trend of topics over time. Additionally, they applied time-to retweet analysis and measure the time between the tweet and the retweets. None of the works discussed in this paragraph have used distributed big data computing platforms such as Apache Spark, supported social media in the Arabic language, or applied spatial analysis.

Mackey et al. [60] applied the Biterm Topic Model (BTM) to detect topics related to COVID-19 symptoms, experiences with access to testing, and disease recovery. They collected around 4 million tweets after filtering by keywords. The data was collected for the period 3 March 2020 to 20 March 2020. Then, the tweets were grouped into five main thematic categories: “conversations about first and secondhand reports of symptoms”, “symptom reporting concurrent with lack of testing”, “discussion of recovery”, “confirmation of negative diagnosis”, and “discussion about recalling symptoms”. For the analysis, they used python packages and R-studio. Additionally, they analyzed the time and location for the geotagged tweets (in our work, we use multiple methods for the location extraction of tweets). Li et al. [61] detected stress symptoms related to COVID-19 in the United States. They integrated a Correlation Explanation (CorEx) learning algorithm and clinical Patient Health Questionnaire (PHQ) lexicon and proposed a CorExQ9 algorithm. They collected 80 million tweets for the period of January 2020 to April 2020 and used a Jupyter computing environment deployed on the Texas A&M High Performance Computer. They compared CorExQ9 with LDA and non-negative matrix factorization (NMF). Moreover, they visualized the symptoms of COVID-19 related stress at the county level for multiple two-week periods. These works differ from our work in multiple aspects, including the differences in the foci of the studies, the overall methodology, the specifics of analysis, the time period of the data used, and particularly the processing of social media in the Arabic language. 

### 2.4. COVID-19 and Twitter (Arabic Language)

We review the works related to COVID-19 analysis that use Twitter data with a focus on the tweets in the Arabic language. Alam et al. [62] analyzed Arabic and English tweets during the COVID-19 pandemic to find whether the tweets contained a factual claim. They defined annotation guidelines for manual annotation. Alshaabi et al. [63] collected tweets in 24 languages including Arabic. They created time series for the top thousand 1 g for each language. Then, they applied basic observations about some of the time series data, including the use of the word “virus” in the tweets of all languages. Alsudais and Rayson [64] collected around 1 million tweets about coronavirus for the period December 2019 to April 2020 and clustered them using the K-means algorithm with the Python Scikit-learn package. They found five topics; these are “COVID-19 statistics”, “prayers for God”, “COVID-19 locations”, “advice and education for prevention”, and “advertising”. Besides this, to identify rumors, they applied supervised classification and labeled 2000 tweets as false information, correct information, and unrelated. The review of the works on COVID-19 analysis using Twitter data in the Arabic language shows that the works on the topic are scarce and are limited in their variety and the depth of the technologies, methods, and analysis used in those works. For example, none of these works have used big data platforms, and none have reported spatio-temporal analysis.

### 2.5. Research Gap, Novelty, and Contributions

The literature review provided in this section clearly establishes the enormous potential of social media analytics for COVID-19-related studies. The traditional methods of data collection and analysis using surveys and other means cannot capture such timely and large-scale data, alongside them having other disadvantages. The state-of-the-art social media analytics for COVID-19-related studies is limited. Many more studies are needed to improve the breadth and depth of the research on the subject with regard to the focus of the studies, the size and diversity of the data, the applicability and performance of the machine learning methods, the diversity of the social media languages, the scalability of the computing platforms, etc. The maturity of research in this area will allow the development, commercialization, and wide adoption of the tools for pandemic-related and general surveillance and other purposes.

**Figure 2:**
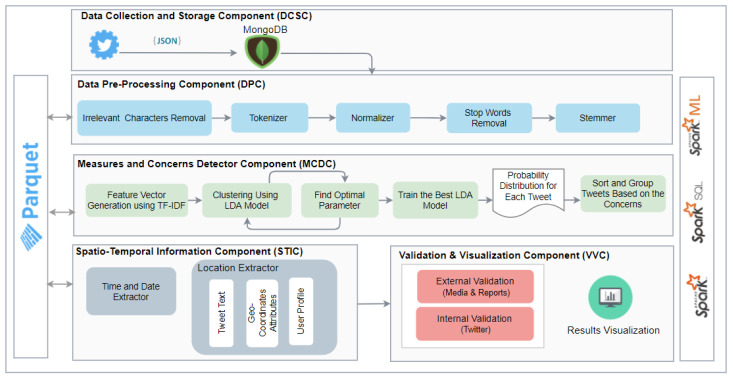
The tool architecture.

The research reported in this paper is different from the existing works on social media analytics for COVID-19-related studies in several respects, including the focus of the studies, the methodology, the size of the data, the time/period of the social media data, support for the social media in the Arabic language, whether the studies have used big data distributed computing platforms, the breadth and the depth of the reported analysis such as spatial and temporal analysis, the geographical focus of the studies, and the specific findings. None of the existing works have reported a similar COVID-19 analysis of Twitter data in the Arabic language with regard to the modelling method used and the depth of the analysis. The Twitter data we have used, its time period, and the methodology of its collection and analysis are different. The methods used for the validation of the findings are also different. None of the existing works on the COVID-19 analysis has used big data technologies for social media in Arabic. Even the works that use big data distributed computing platforms for the analysis of text in languages other than Arabic are very limited and differ in several aspects. The scalability of the software systems for COVID-19 analysis is critical and is being hampered due to the challenges related to the management, integration, and analysis of big data (the 4V challenges). We have developed a novel architecture and pipeline (see Figure 2) for big data management and analysis using distributed machine learning. We have also provided an analysis of the execution time complexity for LDA algorithms for a different number of iterations (between 5 and 1000 iterations) on a varying number of computing cores (see Section 4.4). The use of big data distributed computing technologies is important, because it will allow the scalability and integration of COVID-19-related software with each other and with other healthcare and smart city systems. 

## 3. The System Methodology and Design 

The architecture of the proposed system is depicted in Figure 2. It comprises five components that are depicted in the figure as five separate blocks and discussed in the following subsections subsequent to the overview below.

### 3.1. The System Overview

We built our tool in Apache Spark, which is a big data platform for in-memory computations on distributed data. Apache Spark provides the Spark ML package for machine learning and Spark SQL for data handling. Spark SQL acts as a distributed SQL engine. Additionally, it offers a programming abstraction called DataFrames, which is conceptually equivalent to a table in a relational database but is immutable, parallel, and distributed to handle big data. Moreover, the proposed tool was developed using Python and runs over Aziz supercomputer, which supports running Spark with YARN. Aziz consists of 380 regular computer nodes, 112 compute nodes with large memory, as well as 2 additional GPU compute nodes and 2 additional MIC compute nodes. All the computer nodes run CentOS 6.4 with dual Intel E5-2695v2 processors. Each node has 24 cores. Regular nodes provide 96 GB memory, while large memory nodes provide 256 GB memory. Further, they provided the Fujitsu Exabyte File System (FEFS) which offers high-speed storage to store input/output data for the running jobs. It provides 7 petabytes of memory.

Algorithm 1 shows the master algorithm. The inputs are the search queries and the geo-coordinates, which are required for the Data Collection and Storage Component (DCSC) in addition to the location dictionary, which will be used during spatio-temporal information extraction. The dataset was collected using the Twitter REST API and stored in MongoDB. Then, the tweets will be loaded into Spark DataFrame (DF), which is a distributed data collection organized into named columns. After that, the tweet Dataframe will be passed to the Data Pre-Processing Component (DPC), which removes noise from the text and provides cleaned, normalized, and stemmed tokens. Furthermore, the major concerns will be discovered using the Measures and Concerns Detector Component (MCDC), which applies an unsupervised LDA model to cluster the tweets. Subsequently, to perform spatial and temporal analysis, the date, time, and location information are extracted using a Spatio-Temporal Information Component (STIC). Finally, in the Validation and Visualization Component (VVC), the results are visualized and validated against external or internal sources.
**Algorithm 1:** Master**Input:** search_query; geo_coordinate; location_d **Output:** The discovered concerns and their space and time information**1** *tweets* ← DCSC(search_query, geo_coordinate)**2** *spark* ← createSparkSession()**3** *tweets_DF* ← spark.read(tweets)**4** *tweets_p_DF* ← DPC(tweets_DF)**5** *tweets_g_DF* ←CDC(tweets_p_DF)**6** *tweets_st_DF* ← STEC(tweets_g_DF, location_d)**7** *VVC*(tweets_g_DF, tweets_st_DF)

### 3.2. Data Collection and Storage Component (DCSC)

The experimental dataset contains Arabic tweets collected using Twitter REST API during the period from 1 February to 1 June 2020. The total number of fetched tweets are approximately 14.8 million tweets. The tweets were acquired using two methods. First, we use keywords and hashtags related to coronavirus, such as #corona and #كورونا, #covid19, as well as official accounts that post about it, such as the account of the Saudi Ministry of Health (@SaudiMOH). The second method is fetching tweets without keyword filtering to make sure that we do not miss any important tweets because we want to see what are the topics that people were talking about and how the pandemic has changed their life. Subsequently, we used geolocation filtering to obtain only tweets posted in Saudi Arabia because our main focus in this work is to find the major concerns during the pandemic time in Saudi Arabia. 

Algorithm 2 illustrates the algorithm of the data collection. To store the collected tweets, we searched for a storage method that supports flexible schemas. Therefore, we selected the NoSQL databases, particularly MongoDB, which is a document-oriented database. They enable storing various document data types, such as XML and JSON. 

Moreover, to store the output of each component, we used Parquet file storage. One of the reasons for selecting Parquet is because it is supported by many data processing systems, including Apache Spark. Besides this, it automatically preserves the schema of the original data and provides a good performance for both storage and processing. The files were stored using the Fujitsu Exabyte File System (FEFS), which is a scalable parallel file system based on Lustre. Finally, the duplicated tweets were removed before passing them to the next stage, which is pre-processing.
**Algorithm 2:** Data Collection and Storage**Input:** search_query; geo_coordinate **Output:** The collected tweets**1** *db* ← connect_MongoDB()**2** *api* ← connect_Twitter_API()**3** collect_store_tweetsCOVID19(api, db, search_query, geo_coordinate)**4** collect_store_tweets(api, db, geo)

### 3.3. Data Pre-Processing Component (DPC)

The main pre-processing steps can be summarized as follows: (1) irrelevant character removal, (2) tokenizer, (3) normalizer (4) stop-word removal, and (5) stemmer. In the first step, we removed all the numbers, the English alphabet, and all punctuation marks. This means removing @, which every username started with, and # and _, which are used in hashtags. However, we leave the hashtag name itself if it is not in English because it might include useful information, such as the city name. Removing English and punctuation means also removing links and all punctuation including Arabic semi-colons (؛) and Arabic question marks (؟). Furthermore, we removed the thirteen forms of Arabic diacritics [65] which can be grouped under three categories: vowel, nunation and shadda diacritics. Vowel diacritics include the three main short vowels, called in Arabic Fatha (ـَ ), Damma ( ـُ ), and Kasra ( ـِ ), as well as the Sukun diacritic ( ـْ ), which indicates the absence of any vowel. Nunation diacritics represent the doubled version of the short vowels known in Arabic as Fathatan (ـً ), Dammatan (ـٌ ), Kasratan (ـٍ ). The last form of diacritics is Shadda (germination). It refers to the consonant-doubling diacritical (ـّ ). This also can be merged with diacritics from the two previous types and result in a new diacritic such as (ـُّ ) or ( ـٌّ ). 

The second step is dividing the text into tokens. We used the split() method in Python with the white-space separator. The third step is using the Normalizer to normalize the words (tokens) that contain different forms of Alif (أ, إ, آ), ‘Yaa’ (ي) and ”TAA MARBUTAH/ة” into the basic form. To clarify, the letter “Taa marbutah” (ـة) will be replaced with “haa” (ـه) while “Yaa” (ي) will be replaced with “dotless Yaa” (ى ). Additionally, “Alif” with three forms (أ, إ, آ) will be replaced with “bare Alif” (ا).

The fourth step is removing stop-words. To do this, we modified the stop-words provided by the Natural Language Toolkit (NLTK) to include a new list of stop-words as well as normalize them. Since the NLTK stop-words list was designed for the formal Modern Standard Arabic, we modified the list to include words that usually used in dialectical Arabic, such as “ليش”, “اللي”, “ايش”, and “ليه”, in addition to that we consider the common grammar mistakes. For example, the preposition “على” might be written “علا” and “لكن” might be written “لاكن”. Besides this, we included words that are usually used in Du’aa (prayer) such as “يارب”, “اللهم”, “الله”. After that, we normalized the final stop-words list before using them because they will be extracted from a normalized text. This component is part of our earlier paper, Iktishaf. For further details, see the pre-processing algorithm in [6].

Finally, we stem the tokens using the Iktishaf Light Stemmer [6]. Unlike the existing Arabic light stemmers, Iktishaf stemmer was designed to minimize the number of letters removed and eliminate changes in the meaning. It used a predefined list of prefixes and suffixes. Then, based on the length of the word, the tool decides which affix can be removed. That leads to minimizing the word confusion and losing or changing the word meaning. For further details, see the stemmer algorithm in [6].

### 3.4. Measures and Concerns Detector Component (MCDC) 

To discover concerns, we used the Latent Dirichlet Allocation (LDA) topic modeling algorithm. It is a statistical model that is used to identify the main topics discussed in a collection of documents. It is an unsupervised method that models documents and topics based on dirichlet distribution. Each document is characterized by the probability distribution over various topics while each topic is modeled as a probability distribution over words. The model received a collection of documents and returned a set of topics. Each topic includes a set of words. This required defining the number of topics, denoted by *k* to model the distributions. In this work, the tweets are the documents and we refer to topics as concerns. Apache Spark supports LDA since Spark 1.3.0 in the MLlib package and it also supports it in ML package.

Algorithm 3 illustrates the algorithm of the Measures and Concerns Detector Component. The inputs for this component are the pre-processed tweets (tweet_p). The set concerns number ([K]), the set of iterations number ([R]), and the threshold value. The output of the DPC will be loaded from parquet files and stored in a Spark DataFrame (tweet_DF). For training the model, we need to pass the documents (tweets) as vectors of word counts. Thus, we used the CountVectorizer function. Then, we applied TF-IDF weight, which is a statistical measure used to evaluate how important a word is to a document (tweet) in a collection (tweets). This stands for term frequency-inverse document frequency. TF-IDF comprises of two parts Term Frequency (*TF*) and Inverse Document Frequency (*IDF*). *TF* measures how frequently a word occurs in a tweet. It is calculated using the following equation:

(1) $$\;TF_{w,t}=\frac{f_{wt}}{n_{t}},$$

where *f_wt_* is the frequency of word *w* in tweet *t* and *n_t_* is the total number of words in that tweet.

(2) $$IDF_{t}=1+log\frac{\left|T\right|}{\left|t:w\in t\right|}$$

where |T| is the total number of tweets, and it is divided by the total number of tweets that contain the word *w*. Then, the multiplication of *TF* and *IDF* will represent the weight of the word *w* in tweet *t*.

(3) $$TF-IDF_{w,t}=\;TF_{w,t}\times \;IDF_{t}.$$

After passing the collection of tweets as a vector to the LDA model, we need to specify the number of concerns (*k*), which also can be thought of as cluster centers. To find a suitable number of concerns, we tested different concerns numbers and calculated the perplexity. Perplexity is a statistical criterion of how well a probability model predicts a sample. It is a standard metric to measure generalization performance [66]. Lower perplexity score indicates a good model. Further, we tested different iteration numbers to find the best value.
**Algorithm 3:** Measures and Concerns Detector**Input:** tweets_p; [K]; [R]; threshold **Output:** concerns[][], tweets_g_DF**1** *spark* ← createSparkSession()**2** *tweets_DF* ← spark.read(tweets_p)**3** *features_DF* ← generate_TFIDF_vector(tweets_DF)**4** *LDAmodel* ← get_best_model(LDA_clustering(features_DF, [K], [R]))**5** *concernsProb_tw_DF* ← train_best_model(LDAmodel)**6** *concerns[][]* ← LDAmodel.describeTopics()**7** *concern_tw_DF* ← assign_tweets_to_concern(concernsProb_tw_DF)**8** *tweets_g_DF* ← group_filter_tweets(concern_tw_DF, threshold)

**Figure 3:**
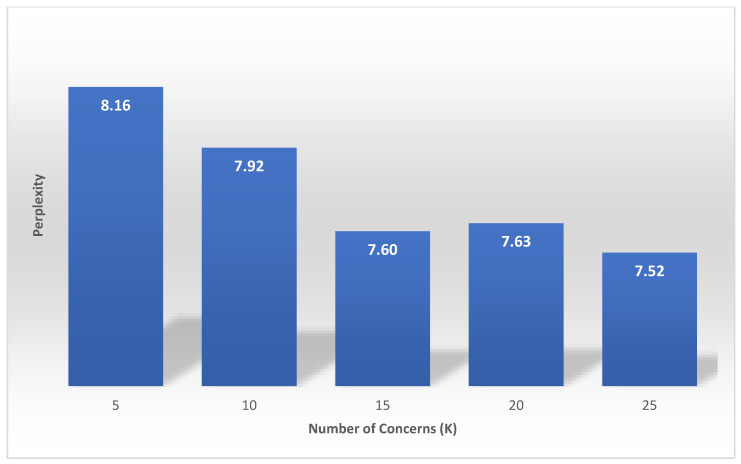
Perplexity score versus the number of concerns.

Figure 3 shows the perplexity score against the number of concerns, *k*. The perplexity score decreases with an increase in the value of *k* with some minor exceptions. The gain in the perplexity score after k=15. is relatively insignificant. Therefore, we use 15 as the value of *k*—i.e., the number of concerns to be detected by our tool is set to 15. We also carried out an empirical analysis of the various concerns detected by different values of *k* and found that k=15 produces the best results.

**Figure 4:**
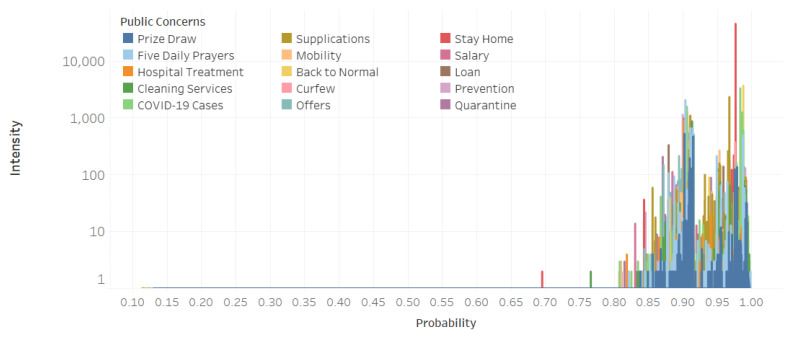
Tweet intensity versus probability of concerns.

Moreover, the model that achieved the best results is trained to obtain a final concerns list. Furthermore, by calling the *describeTopics* function, we obtain a list of the top terms for each concern. From the list of terms, we can understand the concern and thus we define a label that represents it. For each tweet, we get an array of the probability distribution, which represents how much the tweet belongs to each cluster. The concerns probability as well as the tweets are stored in concernsProb_tw_DF. We need to make each tweet belong to one concern (cluster), so we pick the concern with the highest probability in the array and we consider it the best concern that represents the tweet. Thus, we get a group of tweets under each concern. Since we have a large number of tweets, we assume that some of them might be included under a specific concern because it represents the highest probability comparing to the other concerns but the probability value itself might be very low. To keep only tweets that are highly related to the concern, we decide to define a threshold and filter out the tweets that have a probability less than the threshold value. This value will depend on the data; in our particular case, we found that most of the tweets have a probability higher than 0.8 as shown in Figure 4. So, we set the threshold=0.8. The outputs of this component are the lists of top keywords that explain each concern and the tweets grouped by the concerns. The detected concerns will be explained later in the results section (see Section 4). 

In the MCDC component, we also compute the correlation matrix by calculating correlation coefficients between the keywords of the detected concerns. This helps in understanding relationships between the keywords. There are three main types of correlation coefficient formulas, which are Pearson, Kendall, and Spearman correlation. The Pearson correlation coefficient is the most commonly used. We selected it in this work. It measures the linear dependence between two variables. The Pearson correlation between two variables *x* and *y* is computed using Equation (4) below.

(4) $$r=\frac{\sum \left(x_{i}-\bar{x}\right)\left(y_{i}-\bar{y}\right)}{\sqrt{\sum {\left(x_{i}-\bar{x}\right)}^{2}\sum {\left(y_{i}-\bar{y}\right)}^{2}}},$$

where $x_{i}$ is the *i*th value of *x* variable,$\;y_{i}$ is the *i*th value of *y* variable, $\bar{x}$ is the mean of the values of the *x* variable, and $\bar{y}$ is the mean of the values of the *y* variable. 

The correlation matrix (see Section 4.1) is an asymmetrical (K × K) square matrix where AB entry is a cell in the matrix that shows the correlation between two keywords in row A and column B. Each cell has a value between 1 and −1, where 1 represents a strong positive correlation and −1 represents a strong negative correlation. 

### 3.5. Spatio-Temporal Information Component (STIC)

In this work, we identify concerns, and then each tweet under each concern that has information about location or time, we call it an event. To apply spatio-temporal analysis, we need to know the time and the location of the extracted event. 

The obtained data using the Twitter API are encoded using JavaScript Object Notation (JSON). Each tweet object we obtained can have over 150 attributes associated with it according to their documentation [67]. Each child object, such as users and place, encapsulates attributes to describe it. 

We extracted time and date information from “created-at” attribute which shows UTC time when the tweet was created. For location extraction from the tweet object, we applied different techniques. 

The first approach is extracting location names from the “text” attribute. It contains the tweet message. The location name might be explicitly mentioned in the text or it might be part of the hashtags. We generated a dictionary for Saudi cities in English and Arabic as well as their coordinates. Before using the dictionary to search for the cities’ names in the text, we passed the Arabic names list to Iktishaf Light Stemmer because we extracted them from the text after applying pre-processing. However, if the city name is not found in the text, we move to the next approach, which is looking for geo coordinates information. 

Therefore, the second approach is obtaining coordinates from “coordinate” or “place” child objects. The “place” child object includes several attributes, such as “place_type”, “place_name”, “country_code”. The “place_type” can be either city or point of interest (poi). Moreover, we do not move to this approach unless we do not find the information in the text because the associate geo-coordinates within the tweet object represents the location where the user physically present at the time of posting the tweet and it does not necessarily be the actual location of the event that they are talking about. If users disable location services in their smartphones, the value of these attributes will be null.

Thus, the third approach we follow is extracting information from the “user” child object. This contains the user profile information such as the screen name and bio, which includes a short description as well as the country and city name. Users fill in the information manually so they can be written in English or Arabic and they might use different spelling such as Makkah can be written as Makah or mecca. Therefore, our location extractor was designed to extract both English and Arabic names as well as the common names for Saudi cities. However, users usually fill in this information when they create their account and do not change them when they travel to another country/city. That is why we leave this option to the end and we do not apply it unless we do not find the location information from the previous two approaches.

### 3.6. Validation and Visualization Component (VVC)

We followed two methods to validate the identified concerns as well as their spatial and temporal nature. The first method is based on searching against various official sources, reports, and news media on the web. We consider it an external validation. The second method is based on Twitter data we have, where it can give us the detailed information in addition to space and time information, particularly if it was posted by an official news account such as @spagov or the account of Ministry of Health. 

After identifying the public concerns using the MCDC (see Section 3.4), we drew line charts to show changes of concerns overtimes. Further, to show the concerns for their spatial nature, we plotted them on top of the Saudi Arabia map. For this purpose, we used Power BI and Tableau.

## 4. Results and Analysis

We will now discuss the results of our proposed system. Section 4.1 describes the detected pandemic measures and concerns (topics) using LDA. Section 4.2 provides an analysis of the identified measures and concerns as regards their temporal nature (the date) as well as the validation process of the identified concerns using internal sources (Twitter) and external sources (online news media). Section 4.3 provides an analysis in terms of their spatio-temporal nature (the date and the cities). Section 4.4 provides an analysis of the model execution times using distributed computing. Finally, Section 4.5 discusses the relationship between the detected measures and concerns.

### 4.1. COVID-19: Pandemic Measures, Public Concerns, and Macro-Concerns

Table 1 lists the fifteen major pandemic measures and public concerns (hereon we refer to them as public concerns or concerns) discussed by the public on Twitter during the COVID-19 pandemic. These are grouped into six groups that we call macro-concerns (Column 1). These are virus infection, daily matters, contain the virus, social sustainability, economic sustainability, and back to normal. Column 2 gives the rank in terms of the importance of the concern based on the percentage of tweets for each concern (percentage is listed in Column 3). The concerns are listed, firstly, in groups (macro-concerns) and, within each macro-concern, by the descending order of the rank. The fifth column of the table shows the top ten keywords related to each concern. Primarily, these keywords are the clusters extracted by our tool using the LDA approach described in Section 3. Subsequently, we assigned a label (i.e., concern) to each cluster of keywords based on our understanding of the keywords in each cluster. For the purpose of gaining understanding about a cluster of keywords, we looked at the tweets that were associated with a cluster with the highest probabilities (we refer to these as the top-ranked tweets). We illustrate this in the following by example. The first row in the table lists the first public concern, which is **COVID-19 Cases**. This includes keywords including health, announce, new, case, register, and infection. These keywords are usually used by individuals and various organizations (e.g., the Ministry of Health in Saudi Arabia) when disseminating information related to the daily number of cases, deaths, etc. The following is one such tweet by the Ministry of Health (the number of cases, deaths, etc. would vary in these tweets).

*الصحة تعلن عن تسجيل (382) حاله إصابه جديدة بفايروس #كورونا الجديد (كوفيد 19) وتسجل (35) حاله تعافي و (5) حالة وفاه رحمهم الله*

*The Ministry of Health announces the registration of (382) new cases of infection with the new Coronavirus (COVID 19) and records (35) cases of recovery and (5) cases of death, may God have mercy on them.*

The second row lists the concern **Supplications** and its keywords. Supplication is an important part of Muslim beliefs and daily life. Muslims supplicate when they face difficulty or hear good news (they may also supplicate without any good or bad news). To illustrate, Muslims believe that a difficulty is a test from God (Allah), and thus they are encouraged to increase their supplications. During the pandemic, people might pray asking Allah to protect them and others from the virus. Muslims increase their supplications greatly during Ramadhan (the lunar month of fasting that comes once a year). The month of Ramadhan this year (2020) fell between 24 April and 23 May. The keywords for this concern are clearly representative of the label “Supplications”.

The third concern is **Quarantine**. This is one of the methods that have been followed by various countries to prevent the spread of the virus by isolating healthy people from potentially unhealthy people who could have been infected with the SARS-CoV-2 virus. The fourth concern is about the **Five Daily Prayers**. Muslims pray in congregations, next to each other without gaps, at mosques five times a day. The Saudi government suspended all the congregational prayers across all mosques in the Kingdom to prevent the spread of the virus. We found tweets from individuals and organizations similar to the following top-ranked tweet.

*
عاجل ابتداءً من يوم الاحد 8 شوال 1441هـ حتى نهاية يوم السبت 28 شوال 1441هـ السماح باداء الجمعه في جميع مساجد المملكة ماعدا #مكه
*

*Urgent starting from Sunday 8 Shawwal 1441 AH until the end of Saturday 28 Shawwal 1441 AH prayers are permitted to be performed in all mosques of the Kingdom, except for #Makkah*

This explains the existence of the keywords Sunday, Saturday, and Shawwal in the clustered keywords. Shawal is the tenth month of the Islamic lunar calendar. The fifth identified concern is **Stay Home**. From the top keywords, we can see that people consider staying home a strong measure to stop the spread of COVID-19 and save lives. To increase awareness among people about the importance of their role in fighting the coronavirus outbreak, authorities used the slogan “We are all responsible”, which is visible in the keywords of this concern. The sixth concern is **Loan**. The COVID-19 pandemic has severely affected people’s financial situation globally due to reasons such as the loss of jobs. They are seeking loans or struggling to repay loans, which makes it one of the major pandemic concerns. The seventh concern is **Cleaning Services**. During the pandemic, the cleaning services were in high demand such as for cleaning public areas affected by virus-carrying people. The following tweet is an example of this concern.

*#* *امانه_الرياض تواصل جولاتها في تعقيم وتنظيف طرق # الرياض خلال فترة #منع_التجول بهدف توفير بيئة صحية امنه للسكان #واس_عام
*

*#Riyadh* *_municipality continues its tours to sterilize and clean the roads of Riyadh during the period of #curfew to provide a safe and healthy environment for the residents # WAS_general*

The eighth concern is **Hospital Treatment**. From the top keywords, we can see that the need for blood donation became very high during the pandemic. This was an international concern because fewer people donated blood. It could be because they cannot visit hospitals/clinics because of the curfew or because they are worried about getting infected. Besides this, according to the Food and Drug Administration (FDA) [16] the number of blood donations dramatically declined during the pandemic time due to the implementation of social distancing as well as the cancellation of blood drives. We found several tweets in our dataset similar to the following, with differing patient file number and hospital name. We removed the file number from the tweet to protect the patient’s identity.

*
عاجل صاحب الملف ----- بحاجه #تبرع #دم الفصيلة : يقبل جميع الفصائل مستشفى الملك فيصل #جدة
*

*Urgent owner of the file ----- needs #Blood #Donation type: accepts all blood types King Faisal Hospital #Jeddah*

The ninth pandemic-related concern is about the **Prevention** of COVID-19. This is clear from the top keywords: reduce, spread, corona, virus, and others. The top tweets that we found for this concern have shown different prevention strategies applied by the government to instill a sense of responsibility and to increase awareness among people about the importance of their role in fighting the spread of this virus. One of the approaches is enforcing curfew. The following tweet was posted on 22 March by @spagov account, which is the official account of the official Saudi Press Agency (SPA) for the news of the royal decrees, orders, council of ministers, and official statements.

*
خادم الحرمين الشريفين يصدر أمره بمنع التجول للحد من إنتشار # فيروس_كورونا الجديد ابتداءً من الساعة 7 مساءً
*

*The Custodian of the Two Holy Mosques issues a curfew order to limit the spread of the new #Corona_virus starting at 7 p.m.*

Besides this, as another example, the Twitter account of the Ministry of Health (@SaudiMOH) has posted the following tweet on 22 March.

*
من أجل سلامتكم ننصح بتأجيل المواعيد والإجراءات الطبية غير الملحة #الوقاية_من_كورونا
*

*For your safety, we recommend postponing non-urgent medical appointments and procedures. #Coronavirus_prevention*

Another tweet with the same hashtag, #Coronavirus_prevention, was posted by the official account of the Minister of Health Dr. Tawfiq Al-Rabiah (@tfrabiah) on 15 May before the end of the curfew and the return to normal. He encouraged people to wear masks before getting out of their houses.

*
أنصح الجميع بإستخدام الكمامة القماشية عند الحاجة للخروج من المنزل #الوقاية_من_كورونا
*

*I advise everyone to use a cloth mask when going out of the house #Coronavirus_prevention*

Moreover, we found another tweet posted by @SaudiMOH on 30 March about the government order to treat all COVID-19 patients for free.

*
وزير الصحة يعلن عن أمر خادم الحرمين الشريفين يحفظه الله بالعلاج المجاني لجميع المصابين بفيروس #كورونا* *الجديد من المواطنين والمقيمين ومخالفي نظام الإقامة.*

*The Minister of Health announces the order of the Custodian of the Two Holy Mosques, may God preserve him for free treatment to all citizens and residents infected and violators of the residency system with the new #Coronavirus.*

The tenth pandemic-related concern regards **Prize Draw**. Note in Table 1. the top keywords, such as withdrawal, documented, video, gift, and retweet. It is common on social media to see some users announce prizes that will be given to a randomly selected follower who retweets their tweet. This helps them to increase their popularity because they will get more followers and thus it would be a mean of earning. This can be done by individuals or companies. The following tweet is an example.

*
السحب الليلة موثق بالفيديو.. هدية ايفون 11 ريتويت و تابع
*

*Withdrawal tonight is documented in the video … the gift is iPhone 11 retweet and follow*

The 11th public concern includes the keywords roads and traffic, and therefore we named it **Mobility**. The levels of daily mobility have changed significantly during the COVID-19 crisis throughout the world. All forms of transportation from road traffic flow to commercial flight activities have been reduced due to the fear of getting infected and the government lockdowns. The following tweet shows an example from Jeddah, the second largest Saudi city. This was posted on 19 March by the official account of the traffic department in Saudi Arabia, @eMoroor.

*
طرق جده تشهد انخفاضا في مستوى الحركة المرورية ، ممايعكس الالتزام بالاجراءات الوقائيه و الاحترازية شكراً لكم و نتمنى للجميع السلامة
*

*Jeddah roads are witnessing a decrease in the level of traffic, which reflects the commitment to preventive and precautionary procedures [.] Thank you and we wish everyone safety.*

The 12th pandemic-related concern is **Salary**. The top keywords include salary, private, government, and sector. Many employees lost their jobs due to the government lockdown restrictions and the closure of shops. Besides this, small, medium, and large businesses were also severely affected. Many organizations cut down their employees’ salaries and/or laid off their employees. The 13th concern is **Curfew**. The top keywords include prevent, wandering, and the names of some cities. The 14th public concern is **Offers**. Discount, code, and coupon are among the top keywords. Various vendors in order to compensate for their losses due to the business closures in physical spaces have provided offers to attract online shopping customers. 

Finally, the 15th concern is **Back to Normal**. This is related to the issues that need to be addressed for returning to normal life (as opposed to the life during the pandemic). By the end of the curfew, the authorities in Saudi Arabia started a new awareness campaign under the slogan “العودة بحذر” (returning with caution). People were discussing and responding to this campaign on social media. This is the last concern in terms of the ranking, because we believe that it includes fewer tweets compared to the other concerns. The “Back to Normal” was a relatively recent public concern within the dataset this stage had started by the end of May and our dataset contains tweets until 1 June.

**Figure 5:**
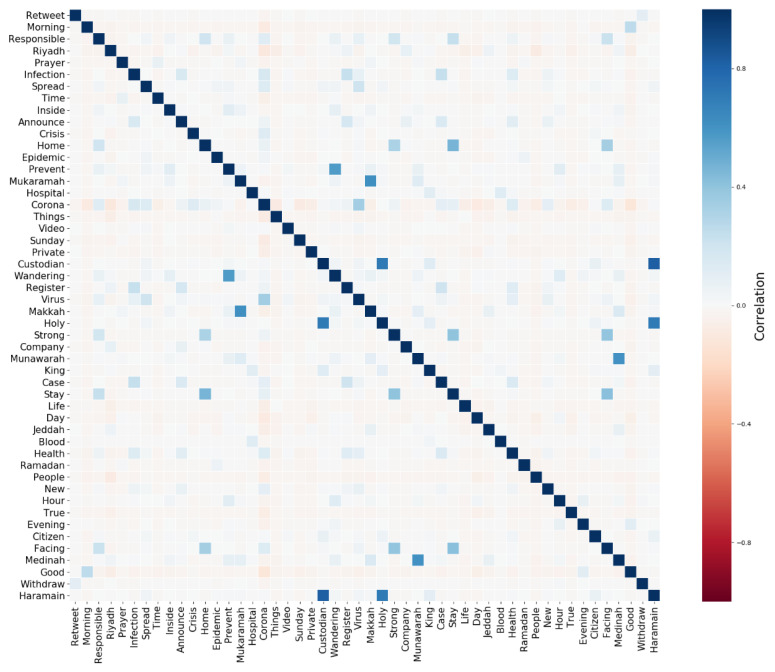
The correlation matrix of keywords.

Figure 5 visualizes the correlation matrix. The correlation matrix is visualized as a heatmap using the Seaborn library in Python. We computed the correlation matrix by calculating the correlation coefficients between the keywords of the detected concerns to show the relationship between the keywords (see Section 3.5 for details on its computations). There are a total of 15 concerns with 10 keywords each. We remove the duplicates keywords that exist in multiple concerns and sort them based on the frequency and keep the top 50 keywords. The dark blue color represents the strongest positive relationship between keywords while the dark red represents the strongest negative correlation. For example, note the dark blue color between wandering and prevent, which are used when mentioning **Curfew**. Note the dark blue squares between the keywords facing, stay, home, and strong, which imply a strong positive relationship between them. As mentioned earlier, these keywords refer to the **Stay Home** concern. There also seems to be a strong positive correlation between custodian, holy, and Haramain, which are usually used when referring to the Custodian of the Two Holy Mosques, the King of Saudi Arabia. Besides, a strong positive correlation can also be noted between Makkah and Mukarramah, which is the full name of Makkah city, as well as Madinah and Munawwarah, which is the full name of Almadinah city, the two holiest cities in Islam. Additionally, note the light blue color between the Makkah and Madinah keywords that shows that these two words have a mild positive relationship, which makes sense because these two cities appear together in many tweets. Note also the positive correlation between case, health, announce, register, corona, and infection. As mentioned earlier, these keywords are used when posting about **COVID-19 cases**.

Note that the most distinctive horizontal or vertical line is the line for the corona keyword, indicating that it has a relatively distinctive relationship with most of the keywords even though the light colors indicate mild positive and negative correlations. The highest positive correlation appears to be between corona and virus, while the highest negative correlation is between corona and good. This makes sense, because good is a positive keyword. Finally, we note that there are not many dark red colors, implying that none of the keywords have strong negative correlations between them.

### 4.2. Temporal Analysis

**Figure 6:**
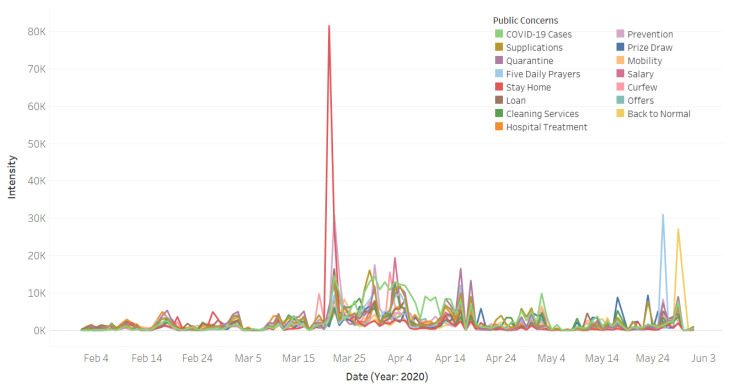
Daily Twitter activity of government measures and public concerns (all).

In this section, we will investigate how the public concerns have evolved over time during the pandemic. Figure 6 depicts the changes in the intensity of the tweets over time for the fifteen identified public concerns. We elaborate the data on these trends in Figure 6 using the following six figures, one for each of the six public macro-concerns.

**Figure 7:**
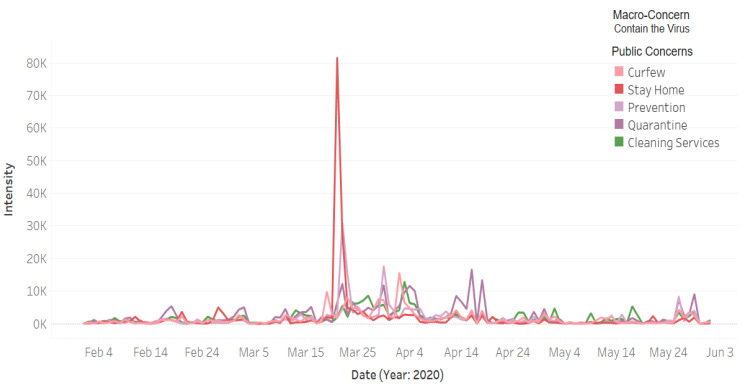
Daily Twitter activity for a macro-concern (**Contain the Virus**).

Figure 7 depicts the intensity of tweets related to the public macro-concern **Contain the Virus**. The public concerns in this macro-class include curfew, stay home, quarantine, prevention, and cleaning services. The curfew was ordered on 22 March and applied from the next day between 7 a.m. and 6 p.m. It can be seen that the highest peak (for **Curfew**) was on 2 April. From external validation [68], we found that on that day the Makkah and Madinah cities were put under a 24 h curfew to prevent the spread of the virus and protect the health of residents. It appears that this 24 h curfew event was this detected highest peak because these are the two holiest cities in Saudi Arabia and for the whole Islamic world, and thus the lookdown of these two cities drew the attention of everyone.

Figure 7 shows the Twitter activity for the **Stay at Home** public concern in red color. It can be seen that the highest peak for this concern was on 21 March. We found that on that day the Government Communication Center of the Information Ministry launched the new visual identity initiative for the awareness campaign for coronavirus under the slogan “كلنا مسؤول” (we are all responsible) to encourage people staying at home [69]. We believe that people interacted with this initiative and posted about it on Twitter using the hashtag #كلنا _مسؤول that explains a large Twitter activity related to the **Stay at Home** concern on that date. The **Prevention** concern is represented in Figure 7 using a light purple color. The highest detected peak for this concern was on 22 March and the second-highest peak was on 30 March. We found that many orders have been placed around the end of March to control the spread of the virus, including the order of curfew that has been announced on 22 March [70]. Further, as posted in the Ministry of Health website, on 30 March the King of Saudi Arabia ordered providing free treatment to all citizens, residents, and even those who violated the residency rules [71]. 

The line plot in purple color in Figure 7 represents the quarantine concern. There are several peaks between 22 March and 18 April. The posts about quarantine had increased after the spread of the virus in the country and the increase in the number of cases. As we mentioned earlier, the government enforced several actions, including lockdown and curfew, as well as closing mosques, schools, and shopping malls by the end of March. The public concern cleaning services is represented in Figure 7 in green color. Note in the graph that the number of tweets start increasing after 22 March and reach the highest point on 3 April. Generally speaking, individuals and organizations have become more careful and concerned with cleanliness. As mentioned in Section 4.1 using example tweets, the Riyadh municipality has been sterilizing and cleaning the roads of the Riyadh city to provide a safe and healthy environment. This tweet was posted on 26 March, which is in the same period that shows a surge in the discussion about this concern.

**Figure 8:**
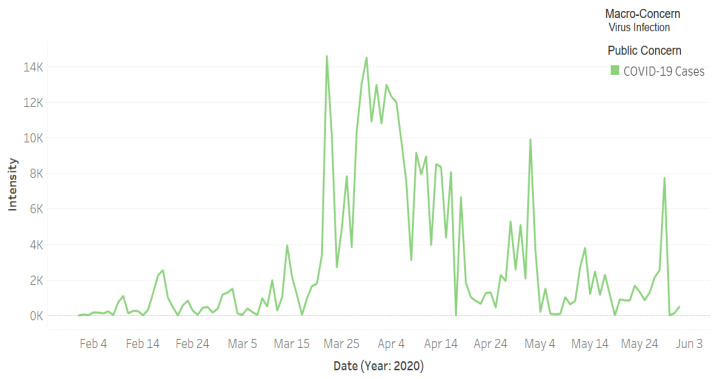
Daily Twitter activity for a macro-concern (virus infection).

Figure 8 depicts the intensity of tweets related to the public macro-concern **Virus Infection** that includes one public concern, **COVID-19 Cases**. Note in the figure that between mid-March 2020 and the end of May (with some intermittent gaps), people have an increased Twitter activity related to the virus infection concern—i.e., the spread of coronavirus and the increase in the number of cases. Specifically, the top two highest peaks are on 22 and 30 March. We found from the external validation process that involves searching in online news media (see Section 3.6) that the number of daily cases increased on 22 March from 48 to 119, while on 30 March the number of cases increased from 96 to 154. This is a significant increase in the number of cases, considering that it was the beginning of the pandemic period in Saudi Arabia. This caught the attention of the people and increased the worries, leading to a peak in the Twitter activity on the subject.

**Figure 9:**
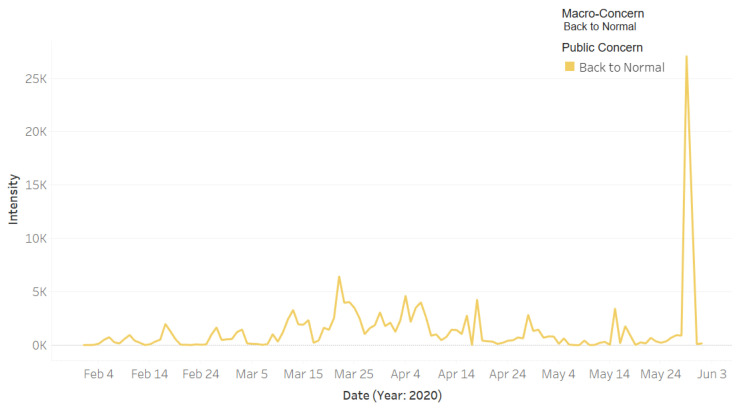
Daily Twitter activity for a public macro-concern (back to normal).

Figure 9 shows the intensity of the tweets for the public macro-concern **Back to Normal** that includes one public concern with the same name **Back to Normal**. The highest peak was on 29 May. We found that on that date the Minister of Health posted the following tweet on Twitter:

*
نحن في بداية أولى مراحل #العودة_بحذر ، لذا اعتمد على التزامكم. إن العودة لزيادة الإحترازات تعتمد على الله ثم على امتثال الجميع. نرجو اتباعكم الإجراءات الوقائية
*

*We are cautiously beginning the first stages of #returning_with_Caution, so we depend on your commitment. We hope that you follow the precautions.*

This tweet was posted by the end of the nationwide coronavirus curfew. The Ministry of Health considered it the first stage to return to normal and started a new awareness campaign under the slogan “العودة بحذر” (returning with caution). The interaction of people with this announcement as well as the use of the hashtag “#العودة_بحذر” explain the increase in the tweet intensity on that day.

**Figure 10:**
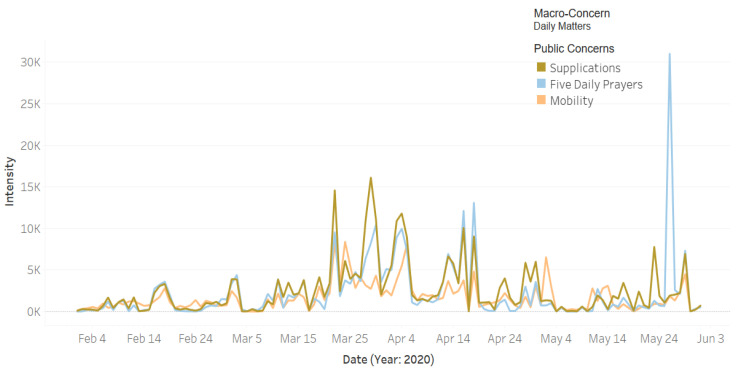
Daily Twitter activity for a public macro-concern (impact on daily life).

Figure 10 plots the intensity of the tweets for the public macro-concern **Daily Matters** that includes three public concerns: **Five Daily Prayers** (**Salah**), **Supplications**, and **Mobility**. Note in the figure that the intensity of the tweets about **Supplications** (see camel color) increased with the spreading of the virus and the increased number of cases. People in Saudi Arabia increased their supplications in response to the COVID-19 crisis. They ask God to protect them and their families from the virus, as well as asking for an end to the pandemic. The light blue color represents the **Salah** concern. The highest peak is on 26 May. Looking in the news media, we found that on that day an official source in the Ministry of Interior announced that, starting from Sunday 8 Shawwal (31 May) until Saturday 28 Shawwal (20 June), they will allow prayers to be held in all mosques of the Kingdom (except the mosques in the Makkah city) [72]. This explains the sharp increase in the tweet intensity on that day, because people were very happy with this news since praying at the mosque is critical for Muslims. The orange color represents the intensity of tweets about the **Mobility** concern. Note in the figure that the highest peak is on 24 March, which is two days after the curfew was implemented in Saudi Arabia. This Twitter activity was in response to how the roads appeared (empty) on the first day of the curfew. We verified this through online articles (see, e.g., [73]). The users of social media shared videos and photos showing the main streets empty due to the coronavirus curfew. 

**Figure 11:**
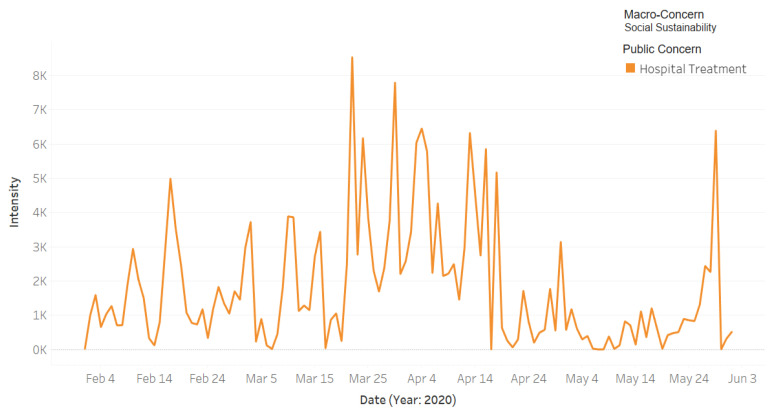
Daily Twitter activity for a public macro-concern (social sustainability).

Figure 11 shows the intensity of the tweets for the public macro-concern **Social Sustainability,** which includes one public concern, **Hospital Treatment**. There was an increase over time in the Twitter activity on this concern during the pandemic, particularly during the later part of March up until mid-April. This was due to the difficulties related to the difficulties in getting treatment at hospitals and other related matters. Particularly, we found several articles in the local newspaper (Okaz) [74,75] encouraging people to donate blood because the blood bank supplies became low due to the COVID-19 situation. Additionally, we found in the collected dataset several tweets about the need for blood donation where they shared the patient files numbers in different hospitals in different cities. Furthermore, the Saudi Twitter hashtags account (@HashKSA) posted the following tweet on 12 April:

*بنوك الدم تشكو قلة المتبرعين بعد جائحة #كورونا.*

*
مديرة بنك الدم في التخصصي “د.الحميدان” تؤكد شدة الحاجة وتحث على التبرع بالدم والصفائح خصوصاً لمرضى #الأورام و #زراعة_الأعضاء
*

*Blood banks complain about the lack of donors after the Corona pandemic. The director of the blood bank in Specialist Hospital, Dr. Al-Humaidan, emphasizes the need and urges to donate blood and platelets, especially for the patients of #oncology and #organ_transplants.*

**Figure 12:**
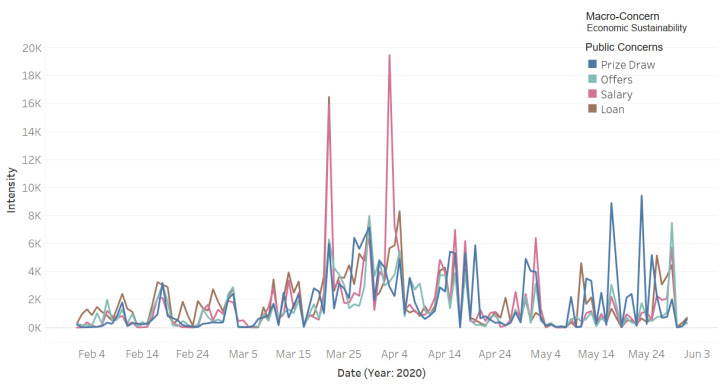
Daily Twitter activity for a public macro-concern (economic sustainability).

Figure 12 depicts the Twitter activity related to the macro-concern economic sustainability, which includes the public concerns **Prize Draw**, **Salary**, **Loan**, and **Offers**. The blue color represents the **Prize Draw** concern. A well-known Twitter activity is about some Twitter users who post about a prize and then pick randomly from users who retweeted their tweet about the prize. One of the reasons for them to do this is to get more followers and become famous, and then this is one of the ways to earn income. This activity helps both the person who wins the prize and the one who announced it. It can be noticed in the graph that, during the pandemic, the intensity of the tweets related to this concern was on the rise. We think that having more free time due to staying at home could be a reason for the increase in such activities on social media. Besides this, the financial difficulties that have become a concern for many people due to the pandemic perhaps have led the people to find other ways to earn income. The green color represents activity for the concern **Offers**. Note in the figure that the intensity of the tweets began to increase around the end of March. The timeline coincides with the timeline of curfew enforcement and shop closures. This, we believe, led business owners to increase sale offers on their products to attract customers to keep shopping from their online stores. Our personal experience in Saudi Arabia in the last few months is that many businesses have gone online or have increased their online sales activities. Social media is one of the free and powerful ways for marketing, and the trend of online shopping and sales offers can be witnessed here. 

The public concern **Salary** in Figure 12 is represented by the magenta color plot in the figure. We found that on 3 April King Salman of Saudi Arabia ordered the government to contribute towards 60% of the salaries of Saudi private-sector employees with a financial incentive of 9 billion Riyals in total [21]. This explains the dramatic rise in the intensity of tweets on that day. The brown color represents the **Loan** concern; its highest peak was on 22 March. We found that on that day the Saudi Arabian Monetary Agency (SAMA) announced that Saudi local banks will postpone the 3-month mortgage installments of all public and private health workers starting from April 2020 [20]. 

### 4.3. Spatio-Temporal Analysis

We investigate in this section the spatio-temporal behavior of selected public concerns during the pandemic. We overlay the location of the specific detected concerns on top of the map of Saudi Arabia. We plot only the tweets that include location information. The size of the circle represents the intensity of the relevant tweets.

**Figure 13:**
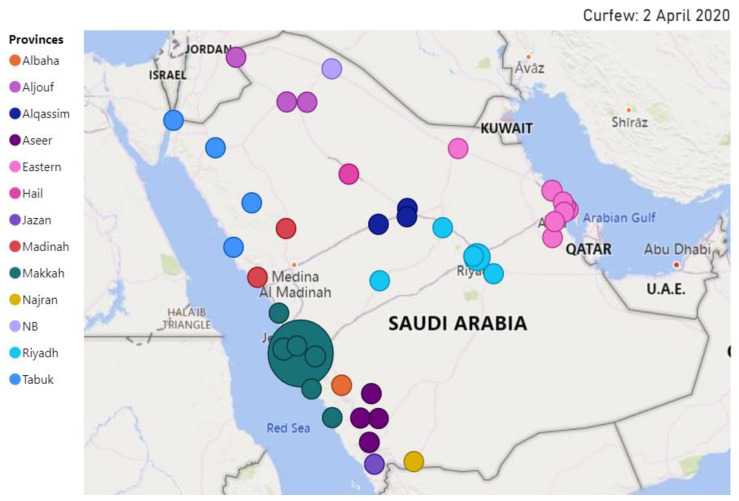
Spatio-temporal behavior of public concern (curfew: 2 April 2020).

Figure 13 depicts the location of tweets about the public concern **Curfew** posted on 2 April 2020. For governance purposes, Saudi Arabia is divided into 13 provinces. Their names are listed on the left of the figure. We have selected the spatial behavior of the concern curfew on this date because the temporal analysis we presented earlier (see Section 4.2, Figure 7) revealed that on that day a 24 h curfew was enforced in the Makkah and Madinah cities. Note in the figure that the largest circle is over Makkah, and this validates the information we already have. We were expecting to find another large circle over Madinah city, but we did not. The official name of Madinah city in Arabic is “المدينة المنورة”, transliterated as “Al-Madinah Al-Munawwarah”. The Arabic word “المدينة” (Al-Madinah) can also mean “the city”, referring to a city that is being referred to in a context, implicitly or explicitly—that is, people may refer to a city as “the city” that is being mentioned in the same tweet or the name of the city may be known from the context of the tweet. The choice we have made in designing the location extractor is that the word “Al-Madinah” if appearing without “Al-Munawwarah” is not considered as a location. We consider the tweet to be about the Madinah city only if the city name is mentioned in full (Al-Madinah Al-Munawwarah). Note in Figure 13 that the activities related to the concern curfew can also be seen in other cities around the kingdom, with some circles (Riyadh) larger than the others. This is because prayers in the main mosques of Makkah (Mecca) and Medina are important for people all around the world.

**Figure 14:**
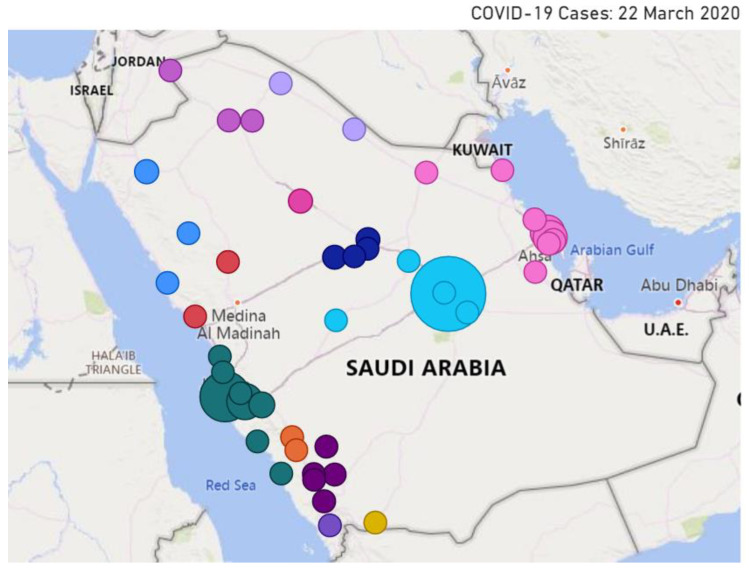
Spatio-temporal behavior of public concern (COVID-19 cases: 22 March 2020).

**Figure 15:**
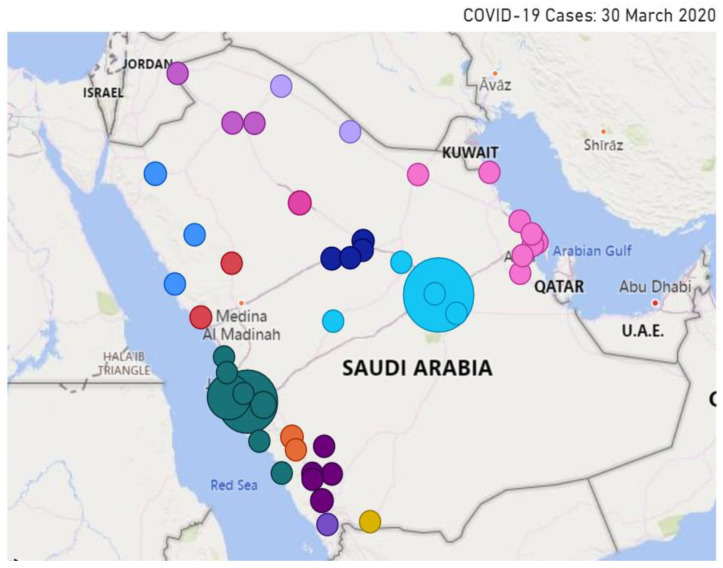
Spatio-temporal behavior of public concern (COVID-19 cases: 30 March 2020).

Figure 14 and Figure 15 illustrate the location of the tweets about the public concern **COVID-19 Cases** on 22 March and 30 March, respectively. These two dates are selected for the concern **COVID-19 Cases** because the temporal analysis we presented earlier (see Section 4.2, Figure 8) has revealed that the two top peak intensities for the concern happened on these two dates. A total of 119 cases were reported on 22 March, 72 of these in Makkah, 43 in Riyadh, 15 in Eastern Province (4 in Dammam, 4 in Qatif, 3 in Alhasa, 3 in Alkhobar, and one in Dhahran), and one in Alqassim [76]. This explains many circles in the eastern province in Figure 14. Each circle represents a city and the size reflects the tweets’ intensity. Note the large light blue circle over Riyadh city and large green circles around Jeddah and Makkah (Jeddah is in Makkah province). We also know that people all around the country were interested in the situation, so they posted about the virus spread and the number of infected people. This explains the presence of circles in different cities around the kingdom.

Figure 15 depicts the spatial information for 30 March. A total of 154 cases were reported on the day with the following distribution: Makkah (40), Dammam (34), Riyadh (22), Madinah (22), Jeddah (9), Haffof (6), Alkhobar (6), Qatif (5), Taif (2), and one in each of the following cities: Yanbu, Buraydah, Alras, Khamis Mushait, Alduwadimi, Dhahran, Samta, Tabuk [77]. To help to understand the map, note that Dammam, Haffof, Alkhobar, Dhahran, and Qatif are in the Eastern Province, whereas the Makkah, Jeddah, and Taif cities are in Makkah Province. Comparing Figure 15 with Figure 14, note that there are some additional circles in Figure 15, implying that the discussion about the public concern had spread to other cities. Moreover, the discussions on the public concern increased in Makkah (dark green circles), perhaps mostly due to the concern becoming a bigger issue over time during March 2020.

### 4.4. Execution Time Analysis

We explained earlier that our tool is designed as a distributed computing tool to address scalability in terms of big data and compute-intensive analytics applications. The tool was developed using the distributed computing platform Apache Spark and was executed on the Aziz supercomputer (see Section 3.1). LDA clustering is RAM-intensive. We have used multiple nodes with 256 GB RAM each.

**Figure 16:**
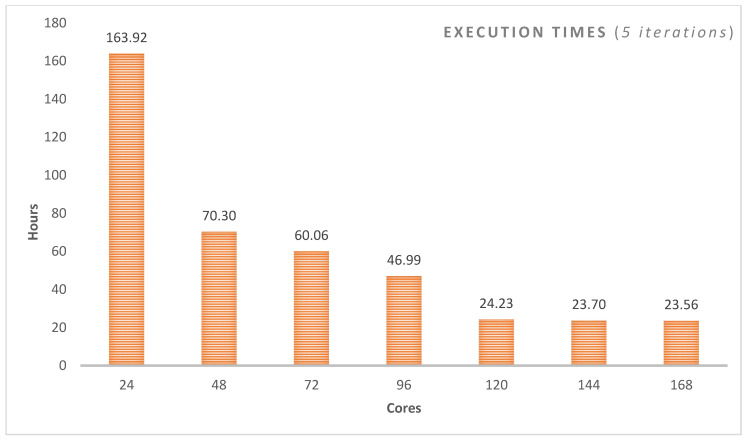
Execution time vs. number of cores for varying number of LDA iterations (no limit on the number of features).

**Figure 17:**
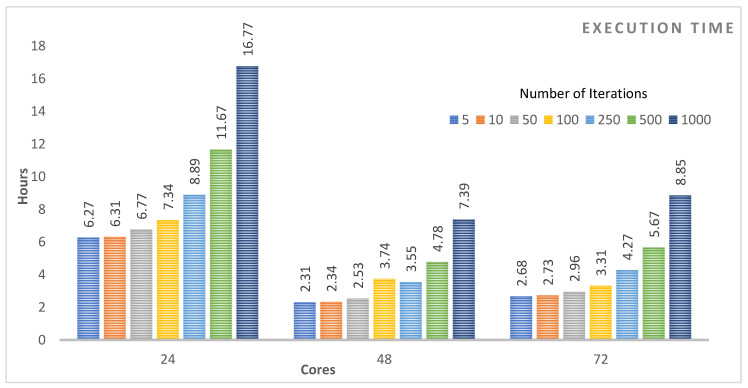
Execution time vs. number of cores for various numbers of LDA iterations (a limited number of features—10,000 keywords).

Figure 16 plots the execution times of the LDA algorithm with five iterations against a varying number of cores (24, 48, 72, 96, 120, 144, and 168). The number of features, in this case, was not limited (compare with Figure 17). The results show that parallelizing the LDA algorithm on a higher number of cores (up to a certain extent) reduces the execution time. The LDA algorithm took 163.9 h (6 days) on 24 cores. We were able to reduce this time to the minimum time of 23.6 h using 168 cores. Increasing the number of cores beyond 120 (to 144 and 168) did not help much and only managed to reduce the execution time of the LDA algorithm a little. This behavior where the execution time of a parallel algorithm does not decrease with an increase in the number of computing cores or nodes is a normal behavior in parallel or distributed computing and happens when the task size is small relative to the number of cores. This is caused by the overhead of parallelizing or distributing a task. Usually, once the parallelization reaches a saturation point where an increase in the number of cores does not decrease the execution time, the execution time may even begin increasing with an increase in the number of cores (see Figure 17). 

Figure 17 plots the execution times against the number of cores (24, 48, and 72) for a varying number of LDA iterations (5, 10, 50, 100, 250, 500, 1000) using 10,000 features/keywords (we have limited the number of features to reduce execution times). For the LDA algorithm with 1000 iterations, we are able to reduce the execution time by more than half, from 16.8 h on 24 cores to 7.4 h on 48 cores, benefitting from an increase in the number of cores. The LDA algorithms with the lower number of iterations (5, 10, 50, …, 500) have also benefited by their execution on a higher number of cores. However, a further increase in the number of cores (72 from 48) does not improve execution speed and rather increases the execution time. As explained earlier, this is a normal behavior in parallel computing due to the parallelization reaching the saturation point.

Generally speaking, a higher number of iterations is expected to produce better clusters. Our experiences in this work suggest that the clusters (public concerns) obtained from 100 iterations were better than the other configurations in terms of the relationship between the keywords of a cluster, etc., enabling us to better label the clusters with appropriate public concern names. Based on the results, the best choice was to execute LDA with 100 iterations on 72 cores. The results reported in this paper are based on this configuration (LDA with 100 iterations and 10,000 features). 

It may appear that the total savings one would obtain by using our tool on Apache Spark would be 4 h (7.34–3.31 h, for the LDA algorithm with 100 iterations). The process of LDA clustering such as presented in this paper may require running the LDA algorithm many times on large volumes of data with different numbers of iterations and features. In our case, we executed the LDA algorithm with various configurations between 30 to 40 times. For this, using the LDA algorithm with 5 to 1000 iterations would easily require over a month of computing time. The ability of the tool to execute in parallel could save a month of computing time in this case and speed up the development process. For larger datasets, executing sequential codes may not even be possible, or distributed computing could save years of development time. How to select the number of cores for a given job that could save experimental time and energy itself is a challenge and has been addressed in our other works [78,79].

### 4.5. Pandemic Measures, and Public Concerns, and Their Interrelationship

Table 1 lists the fifteen major pandemic measures and public concerns discussed by the public on Twitter during the COVID-19 pandemic. The pandemic measures are quarantine, stay home, prevention (COVID-19), cleaning services, curfew, loan, salary, and back to normal. The measures taken by the public and industry to address the economic difficulties caused due to the COVID-19 pandemic are offers and prize draw. The public concerns are COVID-19 cases, supplications, Five Daily Prayers (Salah), mobility, hospital treatment. Some measures, in a way, could also be concerns. For example, quarantine, stay home, prevention (COVID-19), curfew, loan, salary, and back to normal are both measures and concerns. 

The interrelationship or impact of public, industry, or government measures on public concerns can be evidenced in our analysis presented in this section. For example, the events related to loans were being discussed by the public, but were the highest peak was detected on 22 March, the day when the Saudi Arabian Monetary Agency (SAMA) announced it in the media (see [20]). Another example is the “No Mobility” event (empty roads) that was vigorously discussed on 24 March, two days after the curfew measure was announced. The impact of the quarantine and curfew measures was also seen in a reduction in blood donations and blood supplies, leading to increased Twitter activity (concern) on this topic requesting blood donations from late March to mid-April. This concern can also be seen as a measure by the hospital authorities to announce the blood shortage and request action from the public. 

## 5. Conclusions 

The level of digital and physical connectedness of today’s societies has never been seen before. We travel a lot to distant lands and frequently share gifts and viruses with each other. Unfortunately, the COVID-19 pandemic has exposed the vulnerabilities of this unprecedentedly connected world. The COVID-19 pandemic is rapidly growing across the world. Many countries have been affected and the number of cases has greatly increased. World Health Organization (WHO) declared it a pandemic on 11 March 2020. Currently, medical specialists can only treat the symptoms of the disease, since there are no cures for this disease, and developing a new vaccine with low risks and a high success rate will take time. Therefore, it is a serious global health issue. 

Social networking platforms such as Twitter streams hundreds of millions of posts daily. They can be treated as a useful medium for the dissemination of information about diseases. This provides us a great opportunity to study and capture the dynamics of real-world events and understand the various public measures being undertaken by governments, as well as the changes in the daily activities of people during such outbreaks. 

In this paper, we proposed a software tool that aims to detect government pandemic measures and public concerns during the COVID-19 pandemic. The methods used in the tool include an unsupervised Latent Dirichlet Allocation (LDA) topic modeling algorithm, natural language processing (NLP), correlation analysis, and other spatio-temporal information extraction and visualization methods. The tool is built using a range of technologies, including MongoDB, Parquet, Apache Spark, Spark SQL, and Spark ML. The tool, its architecture, five software components, and its algorithms are described in detail. Using the tool, we collected a dataset comprising 14 million tweets from the Kingdom of Saudi Arabia (KSA) for the period 1 February 2020 to 1 June 2020. We formulated and analyzed the findings of this paper from three relationship perspectives: information-structural, temporal, and spatio-temporal.

Concerning the information-structural or subject matter perspective, we have detected 15 government pandemic measures and public concerns and have grouped them into six macro-concerns. For the **pandemic measures** implemented by the Saudi government concerning the COVID-19 pandemic, we detected curfew and restrictions on mobility in the country, quarantine and fines, restrictions on praying in the mosques, campaigns to stay home, COVID-19 prevention, and cleaning services provided to curb the coronavirus spread. For **economic sustainability**, we detected that the government provided financial incentives including loans and private-sector salaries. Businesses increased offers to increase their sales. People moved to or increased in their online economic activities, such as activities related to prize draws for income earnings. For health, well-being, and **social sustainability**, we detected that blood donation and treatment at hospitals have been a major cause of concern. People also actively talked about the new number of cases. The **daily livelihood** issues in Saudi Arabia include the five daily congregational prayers at the mosques that were suspended by the government. People also increased in supplications for the safety of people. A significant reduction in mobility was noted across the country that was related to **environmental sustainability**, health, and well-being due to the reduction in traffic congestion and air pollution. As regards the temporal perspective, we were able to detect the timewise progression of events from the public discussions on COVID-19 cases in mid-March to the first curfew on 22 March, financial loan incentives on 22 March, the increased quarantine discussions during March–April, the discussions on the reduced mobility levels from 24 March onwards, the blood donation shortfall from late March onwards, the government’s 9 billion SAR salary incentives on 3 April, lifting the ban on five daily prayers in mosques on 26 May, and finally the return to normal government measures on 29 May 2020. For the **spatio-temporal** perspective, we extracted location information using different approaches including tweet text and hashtags, geo-coordinate attributes, and user profiles. We were able to detect important events in over 50 cities around the kingdom, with major activities related to COVID-19 cases, curfew, etc., in the Makkah, Riyadh, and Eastern provinces. We validated the detected government measures and public concerns and their spatial and temporal nature through external validation by searching online news media or internal validation by checking tweets.

The detected events in KSA are also aligned with **international concerns,** such as various lockdown measures [14], reduced mobility [15], reduction in blood donations [16], financial difficulties and related government incentives [17,18], and worries related to returning to normal times [19]. Saudi Arabia has followed different strategies to fight the outbreak, instill a sense of responsibility, and raise awareness among people about the importance of their role in the fight against coronavirus. The government undertook early actions to prevent the spread of the virus. KSA reported its first case of the COVID-19 on 2 March. One week later, they closed the schools. On 16 March, they suspended all international and national flights, closed shopping malls, and suspended all sports activities. On 18 March, the attendance of employees at their workplaces in government agencies and the private sector was suspended. Furthermore, the king ordered free treatment for all citizens and residents, even for the violators of the residency system. The KSA government also provided financial incentives in terms of private-sector salaries and the temporary postponement of loan payments.

The research reported in this paper is different from the existing works on social media analytics for COVID-19-related studies in several respects, as has been discussed in detail in Section 2. None of the existing works have reported a similar COVID-19 analysis of Twitter data in the Arabic language in terms of the modelling methods used and the depth of the analysis. The software developed for this work is part of the tool Iktishaf [6,7,8,9] that we have been developing for the last few years. The ability of the tool to execute in parallel could save a month of computing time for the specific dataset size and the problem addressed in this paper and speed up the development process. For larger datasets, executing sequential codes may not even be possible, or distributed computing could save years of development time. 

The findings presented in this paper show the effectiveness of the Twitter media in detecting important events, government measures, public concerns, and other information in time, space, and information-structure with no earlier knowledge about them. The utilization possibilities of such tools are unlimited. For example, governments could learn about the various public concerns in pandemic and normal times and develop policies and measures to address these concerns. The public could raise their concerns and give feedback on government policies. The public could learn about various public and industry activities (such as economic activities detected by our tool) and get involved in these to address financial, social, and other difficulties. The standardization and adoption of such tools could lead to real-time surveillance and the detection of disease outbreaks (and other potentially dangerous phenomena) across the globe and allow governments to take timely actions to prevent the spread of diseases and other disasters. The international standardization of such tools could allow governments to learn about the impact of policies of various countries and develop best practices for national and international response. 

While we have shown good evidence of the use of LDA, NLP, and other methods, more work is needed to improve the breadth and depth of the work with regard to what can be detected, the diversity of data and machine and deep learning methods, the accuracy of detection in space and time, and the real-time analysis of the tweets.

Our focus in this work is on Saudi Arabia. The tool hence currently works with tweets only in the Arabic language. The tool can be used in other Arabic language-speaking countries, such as Egypt, Kuwait, Bahrain, and UAE. The system methodology and design of the tool developed in this paper are generic, and therefore the tool can be extended to other countries globally. This will require the adaptation of the tool with additional languages, such as English, Spanish, or Chinese, by additional modules in the pre-processing and clustering modules.

## Figures and Tables

**Table 1:** COVID-19 pandemic: government measures and public concerns (1 February–1 June 2020).

Macro-Concern	Rank	%	Public Concern	Keywords
**Virus Infection**	1	10.46%	COVID-19 Cases	حاله, اصابه, جديده, حالات, بفيروس, وتسجيل, تسجيل, صحه, جديد, تعلن Case, Infection, New, Cases, Virus, Register, Health, New, Announce
**Daily Matters**	2	8.86%	Supplications	امين, عظيم, بلد, عافيه, مسلم, سبحان, رمضان, وباء, رحمه, اجعل Ameen, Great, Country, Health, Muslim, Glory to, Ramadan, Epidemic, Mercy, Make
	4	7.87%	Five Daily Prayers (Salah)	احد, حياه, شوال, سلام, نهايه, جمعه, صلاه, جماعه, مساجد, سبت Sunday, Life, Shawwal, Peace, End, Friday, Prayer, Group, Mosque, Saturday
	11	5.81%	Mobility	جده, رياض, طريق, عبر, مرور, طرق, وقت, محلى, ارض, حسب Jeddah, Riyad, Road, Through, Traffic, Roads, Time, Local, Land, According to
**Contain the Virus**	3	8.38%	Quarantine	ناس, شيء, حجر, شخص, طيب, حمدلل, يعنى, ممكن, راح, صح People, Things, Quarantine, Person, Fine, Praise be to God, Means, Possible, Go, True
	5	6.53%	Stay Home	مسؤول, فايروس, منزل, كورونا, لمواجه, بقاء, اقوى, سلاحنا, دوره, صحه Responsible, Virus, Home, Corona, Facing, Stay, Strong, Weapon, Circle, Health
	9	6.13%	Prevention (COVID-19)	حرمين, شريف, خادم, ملك, انتشار, مساء, فيروس, كورونا, ساعه, للحد Haramain, Holy, Custodian, King, Spread, Evening, Virus, Corona, Hour, Reduce
	7	6.46%	Cleaning Services	رياض, شركه, تنظيف, مكه, وسلم, مدينه, نقل, امانه, تواصل, امطار Riyadh, Company, Clean, Makkah, Peace, Medinah, Move, Municipality, Continue, Rains
	13	5.70%	Curfew	منع, مكه, تجول, مكرمه, مدينه, داخل, قرار, جبيل, فيديو, منوره Prevent, Makkah, Wandering, Mukaramah, Medinah, Inside, Order, Jubail, Video, Munawarah
**Social Sustainability**	8	6.39%	Hospital Treatment	خير, مستشفى, بحاجه, ملف, صباح, فصيله, دم, تبرع, رقم, صاحب Good, Hospital, Need, File, Morning, Type, Blood, Donation, Number, Owner
**Economic Sustainability**	6	6.48%	Loan	رياض, صباح, حمد, قروض, سداد, يوم, نور, قادمه, امن, اهلى Riyadh, Morning, Thank, Loans, Pay, Day, Light, Coming, Security, My family
	10	5.92%	Prize Draw	كورونا, فيديو, رتويت, هديه, فيروس, سحب, مواطن, يوم, شروط, موثق Corona, Video, Retweet, Gift, Virus, Withdraw, Citizen, Day, Terms, Documented
	12	5.80%	Salary	خاص, تم, قطاع, سعودي, رواتب, حكومه, ازمه, اجتماع, نظام, مجلس Private, Done, Sector, Saudi, Salary, Government, Crisis, Meeting, System, Council
	14	4.74%	Offers	خصم, كود, عكاظ, تكون, اولا, كورونا, وتسجل, نون, كوبون, حقوق Discount, Code, Okaz, Be, First, Corona, Register, Noon, Coupon, Rights
**Back to Normal**	15	4.48%	Back to Normal	عوده, بدايه, اولى, رساله, دفاع, مراحل, احترازه, كورونا, امتثال, تعتمد Back, Beginning, First, Message, Defense, Phase, Precaution, Corona, Compliance, Depend

## Data Availability

Data was obtained from Twitter. Restrictions apply to the availability of these data.
